# Clinical Response to Personalized Exercise Therapy in Heart Failure Patients with Reduced Ejection Fraction Is Accompanied by Skeletal Muscle Histological Alterations

**DOI:** 10.3390/ijms20215514

**Published:** 2019-11-05

**Authors:** Tatiana A. Lelyavina, Victoria L. Galenko, Oksana A. Ivanova, Margarita Y. Komarova, Elena V. Ignatieva, Maria A. Bortsova, Galina Y. Yukina, Natalia V. Khromova, Maria Yu. Sitnikova, Anna A. Kostareva, Alexey Sergushichev, Renata I. Dmitrieva

**Affiliations:** 1 National Almazov Medical Research Centre, 197341 Saint-Petersburg, Russia; tatianalelyavina@mail.ru (T.A.L.); vicka.galenco@yandex.ru (V.L.G.); astroksana@gmail.com (O.A.I.); komarovamy96@yandex.ru (M.Y.K.); lefutr@mail.ru (E.V.I.); marja_@mail.ru (M.A.B.); khromova1610@yandex.ru (N.V.K.); sitnikova@almazovcentre.ru (M.Y.S.); akostareva@hotmail.com (A.A.K.); 2 International Laboratory “Computer Technologies”, ITMO University, 197101 St. Petersburg, Russia; alsergbox@gmail.com; 3 Peter the Great St. Petersburg Polytechnic University, 195251 Saint-Petersburg, Russia; 4 Pavlov First State Medical University of St. Petersburg, 197022 Saint-Petersburg, Russia; pipson@inbox.ru; 5 Department of Women’s and Children’s Health, Karolinska Institutet, 17177 Stockholm, Sweden

**Keywords:** heart failure, exercise training, skeletal muscle wasting, skeletal muscle histology, lactate threshold

## Abstract

Heart failure (HF) is associated with skeletal muscle wasting and exercise intolerance. This study aimed to evaluate the exercise-induced clinical response and histological alterations. One hundred and forty-four HF patients were enrolled. The individual training program was determined as a workload at or close to the lactate threshold (LT1); clinical data were collected before and after 12 weeks/6 months of training. The muscle biopsies from eight patients were taken before and after 12 weeks of training: histology analysis was used to evaluate muscle morphology. Most of the patients demonstrated a positive response after 12 weeks of the physical rehabilitation program in one or several parameters tested, and 30% of those showed improvement in all four of the following parameters: oxygen uptake (VO2) peak, left ventricular ejection fraction (LVEF), exercise tolerance (ET), and quality of life (QOL); the walking speed at LT1 after six months of training showed a significant rise. Along with clinical response, the histological analysis detected a small but significant decrease in both fiber and endomysium thickness after the exercise training course indicating the stabilization of muscle mechanotransduction system. Together, our data show that the beneficial effect of personalized exercise therapy in HF patients depends, at least in part, on the improvement in skeletal muscle physiological and biochemical performance.

## 1. Introduction

Heart failure (HF) is one of the most life-threatening conditions in cardiovascular patients in the world. In chronic heart failure (CHF), functional and metabolic alterations are detected not only in cardiac muscle but also in skeletal muscle tissue [1]. Oxidative stress, systemic inflammation, tissue hypoxia, decreased fatty acid oxidation, and mitochondrial dysfunction are the factors contributing to HF-induced muscle damage [1,2,3]. Pathological skeletal muscle changes include a shift in fiber type, induction of atrophy, development of insulin resistance, dysregulation of lipid metabolism, and ectopic fat deposition. All these factors lead to significant loss of skeletal muscle mass, a decrease in quality of life, and poor prognosis in CHF patients. 

The development of preventive and therapeutic strategies against muscle wasting remains an unresolved challenge. Physical exercise is one of the most common approaches for the treatment of CHF-induced disorders. Currently, aerobic physical training is recognized as an effective and safe therapeutic tactic to achieve stabilization of HF, and exercise training alone or with nutritional support is the most proven strategy to reduce skeletal muscle wasting in CHF patients and is recommended by treatment guidelines [4,5]. Usually, physical training increases the mass of skeletal muscle tissue through the contractility-induced satellite cell activation [6]. In our recent work, we showed that the regeneration potential of bone marrow and skeletal muscle resident stem cells in CHF patients was not severely affected by disease, and under standardized in vitro conditions, these cells maintained proliferation activity [7] and differentiated actively into myotubes [8]. This indicates that exercise-induced activation of the regeneration potential of skeletal muscle stem cells might contribute to muscle tissue restoration and better performance in CHF patients. 

An important issue in the prescription of a therapeutic training program is the determination of exercise intensity optimal for an individual patient. Both anaerobic threshold (AT) and peak oxygen uptake (VO2) are established parameters of exercise tolerance and are known as indicators of severity and prognosis of CHF [9,10,11,12,13] The anaerobic threshold is defined as the exercise intensity before blood lactate concentration becomes too high and begins to accumulate faster than the muscles can remove it, so the intensity is no longer sustainable. Therefore, the lactate (anaerobic) threshold gives an understanding of how the muscles utilize available oxygen, being a more informative indicator of overall athletic performance compared to the VO2 peak [13,14]. We have previously shown that VO2 at lactate threshold and pH threshold might be used as a significant diagnostic and prognostic marker in HF patients [15], and that the prescription of individualized aerobic exercise program based on the definition of lactate threshold resulted in significantly better therapeutic outcome (VO2 peak, left ventricular ejection fraction, exercise tolerance) than in patients who undergo aerobic training prescribed based on the estimation of the VO2 peak [16,17].

In the current work, we utilized AT as a key parameter to establish personalized exercise intensity for each CHF patient and evaluated the exercise-induced clinical outcome along with muscle transcriptome analysis to identify the signaling pathways responsible for modulation of skeletal muscle function in CHF patients undergoing a personalized program of physical rehabilitation. 

## 2. Results

### 2.1. Description of Patients’ Cohort and the Effects of Personalized Training on Clinical Parameters

**Figure 1:**
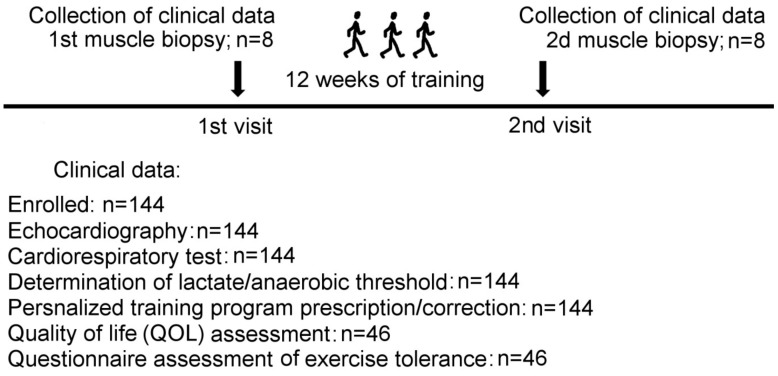
Study design. Skeletal muscle biopsies were taken twice from a selected group of HF patients enrolled in the personalized exercise training program as indicated: Before and after 12 weeks of training. The portion of the first biopsy was used to evaluate the regeneration potential of HF-derived skeletal muscle progenitor cells as we described recently [8]; the rest was flash frozen and saved for further histology analysis.

The study included 144 heart failure patients New York Heart Association (NYHA) III class who underwent personalized aerobic exercise programs based on the estimation of the lactate threshold. The study design is given in Figure 1. Clinical data are presented as metanalysis performed on the whole cohort of HF patients, including those described earlier [15,16,17,18]. All CHF patients enrolled in this project were NYHA class III, in most cases of ischemic etiology, were on stable individually adjusted medical therapy regimes, including angiotensin-converting enzyme inhibitors or angiotensin II receptor antagonists (100%), diuretics (100%), beta-blockers (100%). None of them was diagnosed with diabetes. The baseline clinical characteristics of all patients enrolled in the study are summarized in Table 1. The baseline characteristics of patients’ provided biopsy samples are presented in Table 2.

### 2.2. Physiological Response To Exercise

**Figure 2:**
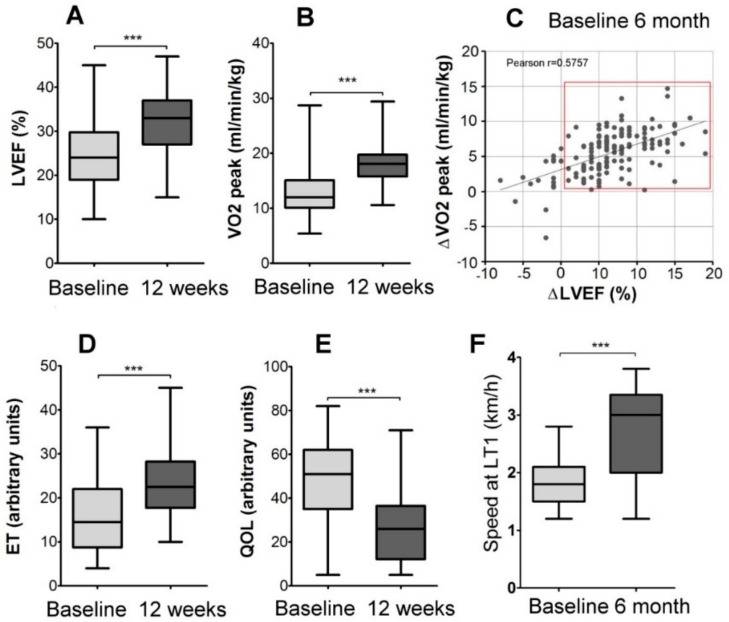
The effects of personalized training on patients’ functional capacity: (**A**) Left ventricular ejection fraction (LVEF) increased significantly after 6 months of personalized training program (*** *p* < 0.0001; *n* = 144); (**B**) oxygen uptake (VO2) peak substantially increased after 12 weeks of personalized training program while VO2 at lactate threshold remained unchanged (*** *p* < 0.0001; *n* = 144); (**C**) the plot of VO2 peak changes versus changes in LVEF after 12 week training course indicates that most of the patients in the group demonstrated an improvement in both parameters (red square); (**D**) exercise tolerance (ET) significantly improved (the rise in arbitrary units indicate the increase in ET; *** *p* < 0.0001; *n* = 46); (**E**) quality of life questionnaire (QOL) demonstrated QOL improvement (the decrease in arbitrary units indicates the increase in QOL; *** *p* < 0.0001; *n* = 46); (**F**) speed at lactate threshold increased significantly after 6 months of personalized training; (*** *p* < 0.0001; *n* = 24).

Before the start of the training program, the lactate threshold (LT1) was determined for each patient as described [19], and a personalized rehabilitation program was prescribed that included 60 minutes of walking speed close to the LT1 4 to 5 times a week. At baseline and after 12 weeks of personalized exercise program, a substantial increase in VO2 peak (cardiorespiratory test), left ventricular ejection fraction (LVEF), exercise tolerance (ET), and quality of life (QOL) were detected (Figure 2A–E). Most of the patients (95%) demonstrated a positive response to the physical rehabilitation program in one or several parameters tested, and 30% of those showed improvement in all four of the following parameters: VO2 peak, LVEF, ET, and QOL. In 11% of patients, LVEF increased up to 40% or more; most of those patients were upgraded to the NYHA II class.

The walking speed at LT1 measured at baseline and after six months of training showed a significant rise in the subgroup of patients (Figure 2F), which clearly indicates the long-term positive physiological response to exercise training program in this group of patients.

**Figure 3:**
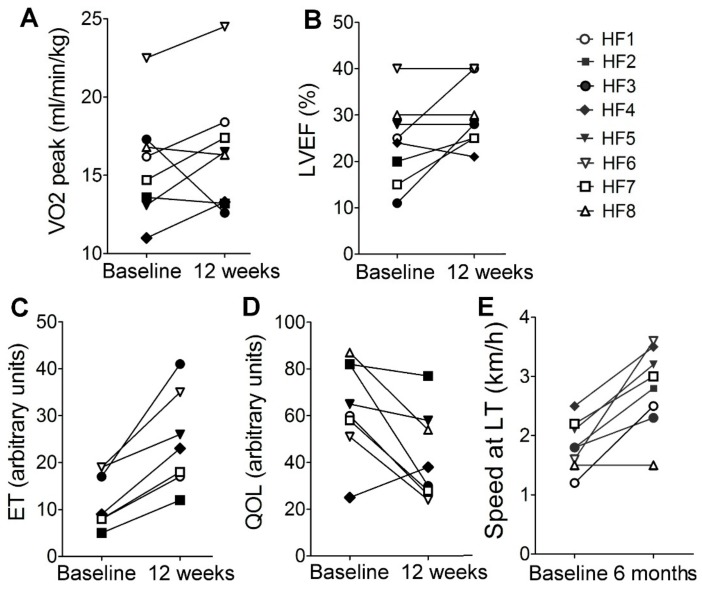
Physiological parameters of patients who provided skeletal muscle biopsy for histological and transcriptome analysis before and after exercise training program: (**A**) cardiorespiratory test (VO2 peak), (**B**) left ventricular ejection fraction (LVEF), (**C**) exercise tolerance (ET), (**D**) quality of life test (QOL), (**E**) speed at LT1. The increase in ET value indicates improvement in exercise tolerance; the decrease in QOL value indicates the improvement in the quality of life test results. Patient HF8 did not show up for the ET test.

All patients included in the “muscle biopsy subgroup” also demonstrated substantial improvements during 12 weeks of rehabilitation in at least three out of four parameters tested in this study: ET, VO2 peak, LVEF, and QOL (Figure 3A−D). All but one (HF8) also demonstrated a rise in walking speed at LT1 after 6 months of exercise training (Figure 3E).

### 2.3. Muscle Histology

**Figure 4:**
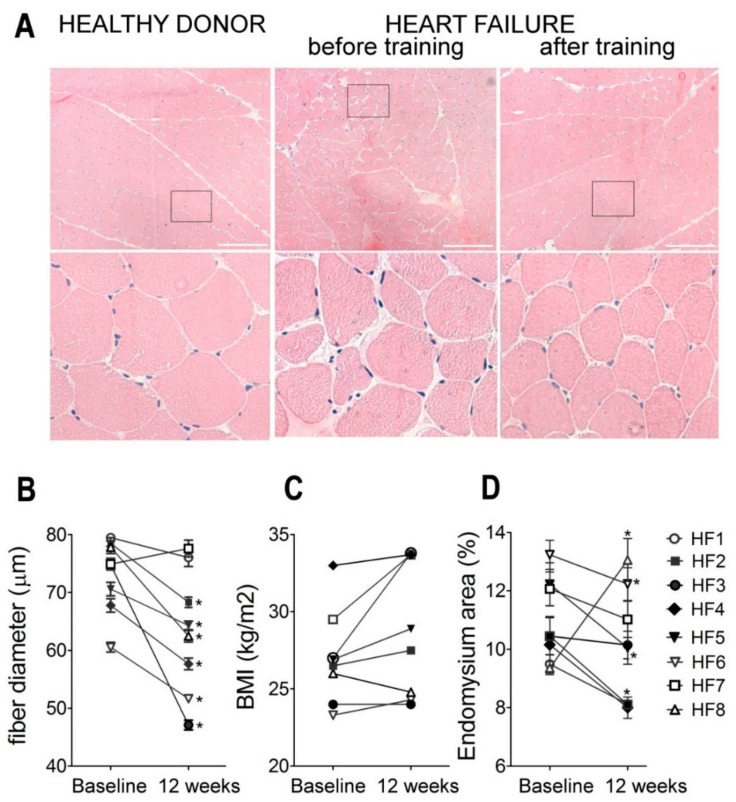
Skeletal muscle histological analysis before and after exercise training. (**A**) Representative histological images before and after exercise training are given for patient HF6. Images of the healthy donor are given for reference purposes. Scale bars represent 200 μm; (**B**) skeletal muscle fiber thickness before and after training in patients with HF (*n* > 60 for each experimental point; * *p* < 0.001; whiskers indicate min and max fiber diameter in the sample; line in the box indicates the median), (**C**) body mass index (BMI) changes after the training course in HF patients in the group; (**D**) individual data for the cross-sectional area of endomysium (*n* > 50 fibers for each experimental point; * *p* < 0.05).

Using biopsy specimens, we analyzed fiber and endomysium thickness in skeletal muscle before and after exercise the rehabilitation program in eight patients comprising the ‘muscle biopsy subgroup’. Conventional morphological examination revealed decreased fiber thickness and accumulation of fibrotic tissue in skeletal muscle of HF patients before training. In six out of eight patients, we detected a significant decrease in muscle fiber thickness after training (Figure 4A,B). The patients HF1 and HF7, who did not demonstrate a decrease in muscle fiber thickness, demonstrated a substantial increase in BMI (Figure 4C). Furthermore, after the training courses in all patients but HF8, a noticeable decrease in the endomysium area was observed (Figure 4A,D). It is important to note here that HF8 was the only patient in the biopsy group who demonstrated a decrease in BMI, along with a simultaneous decrease in fiber diameter and substantial enlargement of endomysium and no effect on speed at LT1 after six months of training.

## 3. Discussion

Normally, training-induced adaptations in healthy subjects are reflected by changes in contractile proteins and function, mitochondrial function, metabolic regulation, intracellular signaling, and transcriptional responses (reviewed in [20]). HF patients have reduced maximum power output. Therefore, the relative load is higher in HF patients than in healthy donors, and this issue should be taken into account when data obtained from healthy donors and HF patients are compared [21]. Furthermore, exercise intolerance in HF patients might be a result of reduced oxidative metabolism and increased gluconeogenesis in the exercising muscle [22] and/or because of low oxygen delivery and physical inactivity of HF patients, or both [21] and these restrictions should also be considered. Therefore, to set the correct personalized exercise intensity, we determined the LT1 [19] for each patient and prescribed 60 minutes of daily walking at speed close to the LT1. The main finding of the present study is that the training-induced health improvements, including the significant increase in speed at LT1 and exercise tolerance in heart failure patients (Figure 2F and Figure 3E), were associated with significant changes in skeletal muscle histology.

In our work, the histological analysis detected a small but significant decrease in both fiber and endomysium thickness (Figure 4) after the exercise training course. It is known that in CHF population, skeletal muscle atrophy is tightly associated with reduced exercise capacity: reduced cardiac output (particularly during exercise) results in reduced skeletal muscle blood flow, muscles do not receive sufficient nutrient supply including oxygen and are either stressed, reversibly injured, or in more extreme conditions undergo apoptotic and/or necrotic cell death [23], resulting in decreased fiber thickness and accumulation of fibrotic and adipose tissue. The exercise training course was expected to improve the morphological characteristics, and the observed decrease in fiber thickness in our patients after exercise training was rather surprising. These observations, however, fit well previous findings by Ades et al. [24] who demonstrated the increase in fiber thickness in CHF patients after 12 months of training, but not after 12 weeks, and by Larsen et al. who demonstrated the improvement in exercise tolerance along with reduction of fiber thickness and increase in capillary density in HF patients after 12 weeks exercise training course [25]. Larsen and co-authors suggested that fiber diameter did not increase during training to maintain a smaller area for the better diffusion of nutrients and gases by capillary networks. Indeed, these cellular changes would result in the ability to sustain sufficient levels of muscle contractions for greater periods without fatigue, explaining the observed increase in exercise tolerance in patients.

The training-induced changes in endomysium observed here also deserve some further discussion: It was reported previously that the immobilization-induced alterations in the skeletal muscle manifested not only in the reduction of fiber length and diameter, but also in the increase and disorganization in the intramuscular connective tissue which disturb the normal structure of the endomysium, contribute to the decreased function, and diminished biomechanical properties of immobilized/disused skeletal muscles [26,27,28]. We suggest that training-induced decrease in endomysium thickness results in stabilization of muscle mechanotransduction system not only at the level of single muscle-fiber but also on the tissue level, thus, contributing to the increase in exercise tolerance.

To summarize, in this work, we have shown that along with clinical response, the personalized exercise therapy in HF results in significant histological alterations in skeletal muscle and that the training-induced beneficial effect depends, at least in part, on the improvement in skeletal muscle physiological and biochemical performance.

## 4. Materials and Methods

### 4.1. Study Population

The study was approved by the Ethics Committee of the Almazov National Medical Research Centre (Ref. # 54; 14/03/2016) and was conducted in compliance with current Good Clinical Practice standards and in accordance with the principles under the Declaration of Helsinki (1989). All patients entering the program agreed to and signed an institutional review board-approved statement of informed consent.

A total of 144 patients (107 male, 37 female) with HF NYHA III class was enrolled. The design of the study is presented in Figure 1. All patients performed a cardiopulmonary exercise test (CPX) and echocardiography and blood sampling. Clinical and laboratory characteristics of all patients and hemodynamic conditions were collected before the beginning of the physical rehabilitation program. Biopsy specimens were collected from a subset of patients (*n* = 8) twice: at the time of enrollment and after 12 weeks of exercise program. The obtained biopsy samples were immediately snap-frozen, placed in liquid nitrogen until final analysis. Three healthy donors were enrolled for reference measurements in histology analysis.

### 4.2. Cardiopulmonary Exercise Testing and Lactate Threshold Determination

A cardiorespiratory test was performed on a treadmill model Охусon Pro (Erich Jaeger GmbH&Co KG, Friedberg, Germany) using a 7 Watt/30 sec ramp protocol [13] for all 144 patients at baseline and after 12 weeks of training. Gas exchange data were collected continuously with an automated breath-by-breath system. During the test, a 12-lead electrocardiogram (ECG) was continuously recorded, and blood pressure was measured every two minutes. Physical activity was stopped if the subject had shortness of breath, fatigue, leg pain at the level of 8 out of 10 on the Borg scale, if blood pressure decreased by 25%, if the patient reported the development of severe weakness, dizziness, or at any other patient request. Blood samples were taken at baseline and then every one minute during the test. To determine AT, the lactate concentration was estimated using analyzer i-STAT, cartridge CG4 (Abbot, Princeton, NJ, USA), as described [29].

### 4.3. Echocardiography

Resting two-dimensional and tissue Doppler echocardiography was performed for all 144 patients at baseline and after 12 weeks of training according to the guidelines of the American Society of Echocardiography 2015 to assess LVEF, morphology, and function. 

### 4.4. Quality of Life and Exercise Tolerance Tests

Quality of life (QOL) and exercise tolerance (ET) were estimated at the baseline and after 12 weeks of physical rehabilitation. Forty-six patients completed both questionnaires successfully. QOL was evaluated by using the Minnesota life heart failure questionnaire (MLHFQ), and ET with the international physical activity questionnaire (IPAQ).

### 4.5. Exercise Therapy Protocol

An individual physical rehabilitation program for HF patients included 1hour of walking at 90% of load intensity at the lactate threshold level (estimated as speed, km/h) for at least 4 to 5 times a week; patients were required to have a diary of self-control (evaluated monthly); the correction of the training load was done on the second outpatient visit; all patients were observed by a cardiologist in the outpatient department. Twenty-four patients, including those who provided biopsy samples, came to estimate the progress in load intensity at LT1 after 6 months of training.

### 4.6. Histology

Histology was assessed on muscle specimens after hematoxylin and eosin staining. Myofiber cross-sectional area was analyzed as described [25]: imaging was acquired using light microscope Axio Observer.Z1 (Carl Zeiss Microscopy GmbH, Jena, Germany) and ZEN Pro/desk software with final magnification × 100. For analysis, only myofibers with symmetrical shape and distinct boundaries were chosen. Size was quantified by measuring the minimal diameter of elliptical-shaped fibers in the transverse section. Diameters were evaluated for 50 ± 20 fibers from at least five microphotographs of an individual sample using AxioVision software. The analysis was performed in a blind manner. 

### 4.7. Determination of Endomysium Area

Within the fascicle, the endomysium is defined as the connective tissue surrounding single myofibers. The endomysium area was calculated as described earlier [30] with some modifications: In the images, we selected areas containing only myofibers and endomysium. For image segmentation and analysis ZEN Intellesis module was used that employs a deep learning platform for image segmentation and analysis. The endomysium area was normalized to the total area of the selected region and presented in percent. The example of images prepared for analysis is given in Figures S1 and S2.

### 4.8. Statistical Analysis

The GraphPad Prism program was used to analyze data; to evaluate the training effects, we used the paired *t*-test. All further details are given in the figure’s legends.

## Figures and Tables

**Table 1:** Baseline characteristics of patients enrolled in the study (*n* = 144).

**Age, years**	53 + 4
**Male/female, *n***	107/37
**BMI, kg/m^2^**	26.6 + 2.5
**NYHA III, *n***	144
**LVEF, %**	26 + 7
**VO2 peak (ml/kg/min)**	12.9 + 3.8
**Disease etiology (DCM/CAD), *n* (%)**	48/96 (67/33)
**ACEI/Beta-blockers/Diuretics (%)**	100/100/100
**Cardiac resynchronization therapy, *n* (%)**	31 (22)
** Coronary artery bypass graft, *n* (%) **	43 (30)
**COPD, *n* (%)**	52 (36)
**Atrial fibrillation, *n* (%)**	18 (12)
**Anemia, *n* (%)**	7 (5)

ACEI, angiotensin-converting enzyme inhibitor; BMI, body mass index; COPD, chronic obstructive pulmonary disease; DCM, dilated cardiomyopathy; CAD, coronary artery disease; LVEF, left ventricular ejection fraction; NYHA, New York Heart Association.

**Table 2:** Baseline characteristics of patients’ provided biopsy samples.

**Donors**	HF1	HF2	HF3	HF4	HF5	HF6	HF7	HF8
**Age, years**	56	48	63	61	56	62	52	54
**BMI, kg/m^2^**	27.07	26.46	24,1	32.87	26.77	23.32	29.5	26.12
**LVEF, %**	25	20	11	24	28	40	15	30
**VO2 peak (ml/kg/min)**	16.2	13.6	17.3	11	13.1	22.5	14.7	28.2
**Disease etiology (DCM/ICM)**	DCM	DCM	CAD	CAD	CAD	CAD	CAD	DCM

All patients were males, NYHA III class, were on stable individually adjusted medical therapy regimes, including angiotensin-converting enzyme inhibitors or angiotensin II receptor antagonists, diuretics, beta-blockers; did not have comorbidities (COPD; CAD; atrial fibrillation; anemia); LVEF, left ventricular ejection fraction; NYHA, New York Heart Association; DCM, dilated cardiomyopathy; CAD, coronary artery disease; COPD, chronic obstructive pulmonary disease.

## Supplementary Materials

Supplementary materials can be found at https://www.mdpi.com/1422-0067/20/21/5514/s1.
