# Supplementary material for: Clinical Response to Personalized Exercise Therapy in Heart Failure Patients with Reduced Ejection Fraction Is Accompanied by Skeletal Muscle Histological Alterations
Source: Int J Mol Sci. 2019 Nov 5;20(21):5514. doi: 10.3390/ijms20215514 (PMC6862491; doi:10.3390/ijms20215514)
Supplement: Supplementary file 1 [file ijms-20-05514-s001.pdf]

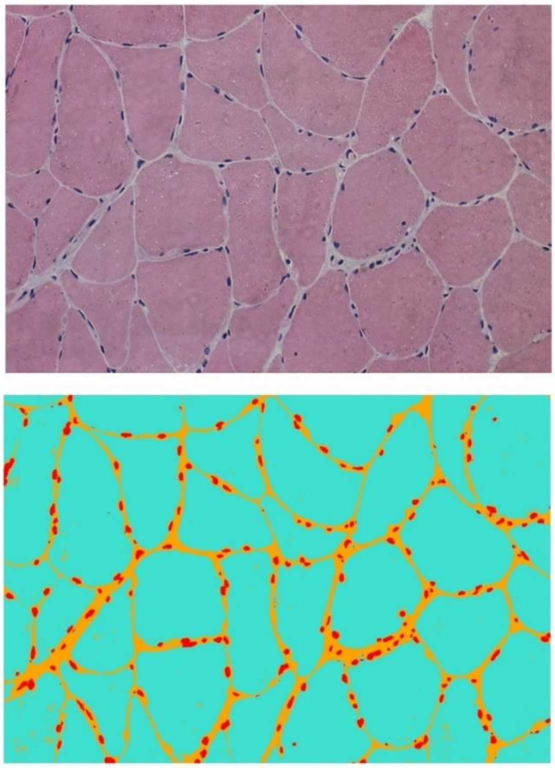

Figure S1. The example of image segmentation for further analysis prepared using ZEN Intellesis module with deep learning platform.

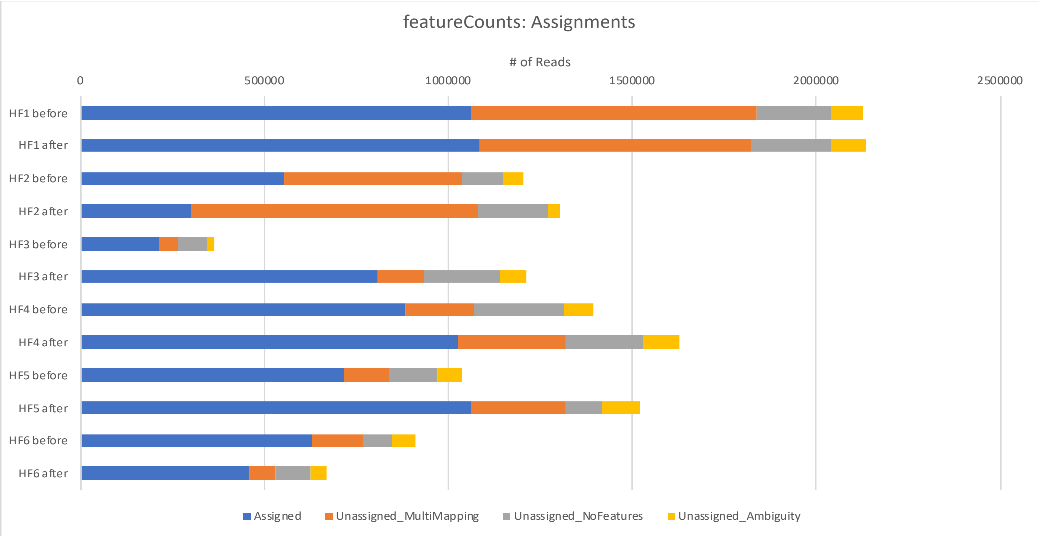

Figure S2: First six patients HF1-HF6 were processed for sequencing. However, HF3 and HF6 samples did not reach sufficient library depth, while HF2 had a low gene assignment rate for the reads
